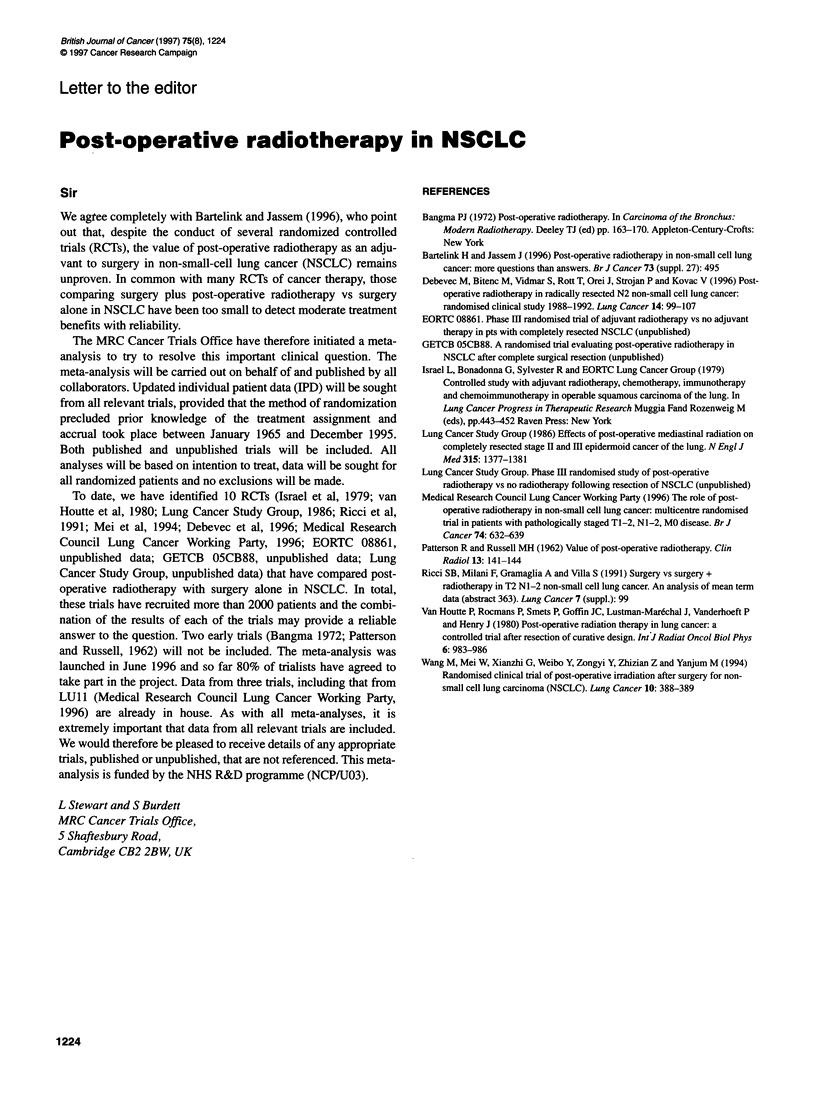# Post-operative radiotherapy in NSCLC.

**DOI:** 10.1038/bjc.1997.210

**Published:** 1997

**Authors:** L. Stewart, S. Burdett


British Journal of Cancer (1997) 75(8), 1224
© 1997 Cancer Research Campaign

Letter to the editor

### Post-operative radiotherapy in NSCLC

### Sir

We agree completely with Bartelink and Jassem (1996), who point out that, despite the conduct of several randomized controlled trials (RCTs), the value of post-operative radiotherapy as an adjuvant to surgery in non-small-cell lung cancer (NSCLC) remains unproven. In common with many RCTs of cancer therapy, those comparing surgery plus post-operative radiotherapy vs surgery alone in NSCLC have been too small to detect moderate treatment benefits with reliability.

The MRC Cancer Trials Office have therefore initiated a meta-analysis to try to resolve this important clinical question. The meta-analysis will be carried out on behalf of and published by all collaborators. Updated individual patient data (IPD) will be sought from all relevant trials, provided that the method of randomization precluded prior knowledge of the treatment assignment and accrual took place between January 1965 and December 1995. Both published and unpublished trials will be included. All analyses will be based on intention to treat, data will be sought for all randomized patients and no exclusions will be made.

To date, we have identified 10 RCTs (Israel et al, 1979; van Houtte et al, 1980; Lung Cancer Study Group, 1986; Ricci et al, 1991; Mei et al, 1994; Debevec et al, 1996; Medical Research Council Lung Cancer Working Party, 1996; EORTC 08861, unpublished data; GETCB 05CB88, unpublished data; Lung Cancer Study Group, unpublished data) that have compared post-operative radiotherapy with surgery alone in NSCLC. In total, these trials have recruited more than 2000 patients and the combination of the results of each of the trials may provide a reliable answer to the question. Two early trials (Bangma 1972; Patterson and Russell, 1962) will not be included. The meta-analysis was launched in June 1996 and so far 80% of trialists have agreed to take part in the project. Data from three trials, including that from LU11 (Medical Research Council Lung Cancer Working Party, 1996) are already in house. As with all meta-analyses, it is extremely important that data from all relevant trials are included. We would therefore be pleased to receive details of any appropriate trials, published or unpublished, that are not referenced. This meta-analysis is funded by the NHS R&D programme (NCP/U03).

*L Stewart and S Burdett*
*MRC Cancer Trials Office,*
*5 Shaftesbury Road,*
*Cambridge CB2 2BW, UK*

### REFERENCES

Bangma PJ (1972) Post-operative radiotherapy. In *Carcinoma of the Bronchus: Modern Radiotherapy.* Deeley TJ (ed) pp. 163–170. Appleton-Century-Crofts: New York

Bartelink H and Jassem J (1996) Post-operative radiotherapy in non-small cell lung cancer: more questions than answers. *Br J Cancer* **73** (suppl. 27): 495

Debevec M, Bitenc M, Vidmar S, Rott T, Orei J, Strojan P and Kovac V (1996) Post-operative radiotherapy in radically resected N2 non-small cell lung cancer: randomised clinical study 1988–1992. *Lung Cancer* **14**: 99–107

EORTC 08861. Phase III randomised trial of adjuvant radiotherapy vs no adjuvant therapy in pts with completely resected NSCLC (unpublished)

GETCB 05CB88. A randomised trial evaluating post-operative radiotherapy in NSCLC after complete surgical resection (unpublished)

Israel L, Bonadonna G, Sylvester R and EORTC Lung Cancer Group (1979) Controlled study with adjuvant radiotherapy, chemotherapy, immunotherapy and chemoimmunotherapy in operable squamous carcinoma of the lung. In *Lung Cancer Progress in Therapeutic Research* Muggia Fand Rozenweig M (eds), pp.443–452 Raven Press: New York

Lung Cancer Study Group (1986) Effects of post-operative mediastinal radiation on completely resected stage II and III epidermoid cancer of the lung. *N Engl J Med* **315**: 1377–1381

Lung Cancer Study Group. Phase III randomised study of post-operative radiotherapy vs no radiotherapy following resection of NSCLC (unpublished)

Medical Research Council Lung Cancer Working Party (1996) The role of post-operative radiotherapy in non-small cell lung cancer: multicentre randomised trial in patients with pathologically staged T1–2, N1–2, M0 disease. *Br J Cancer* **74**: 632–639

Patterson R and Russell MH (1962) Value of post-operative radiotherapy. *Clin Radiol* **13**: 141–144

Ricci SB, Milani F, Gramaglia A and Villa S (1991) Surgery vs surgery + radiotherapy in T2 N1–2 non-small cell lung cancer. An analysis of mean term data (abstract 363). *Lung Cancer* **7** (suppl.): 99

Van Houtte P, Rocmans P, Smets P, Goffin JC, Lustman-Maréchal J, Vanderhoeft P and Henry J (1980) Post-operative radiation therapy in lung cancer: a controlled trial after resection of curative design. *Int J Radiat Oncol Biol Phys* **6**: 983–986

Wang M, Mei W, Xianzhi G, Weibo Y, Zongyi Y, Zhizian Z and Yanjum M (1994) Randomised clinical trial of post-operative irradiation after surgery for non-small cell lung carcinoma (NSCLC). *Lung Cancer* **10**: 388–389